# Endometriosis Classification and The Role of Tumor Necrosis Factor-Alpha Polymorphisms as A Therapeutic Target

**DOI:** 10.22074/ijfs.2020.5876

**Published:** 2020-02-25

**Authors:** Fabio Barra, Lorenzo Ferro Desideri, Umberto Leone Roberti Maggiore, Valerio Gaetano Vellone, Mattia Maramai, Carolina Scala, Simone Ferrero

**Affiliations:** 1 Academic Unit of Obstetrics and Gynecology, IRCCS Ospedale Policlinico San Martino, Genoa, Italy; 2 Department of Neurosciences, Rehabilitation, Ophthalmology, Genetics, Maternal and Child Health (DiNOGMI), University of Genoa, Genoa, Italy; 3 Department of Surgical and Diagnostic Sciences, IRCCS Ospedale Policlinico San Martino, Genoa, Italy

**Keywords:** Endometriosis, Genetic Polymorphisms, Tumor Necrosis Factor-Alpha

## Abstract

In the recent original research published on International Journal of Fertility and Sterility the association between tumor necrosis factor-alpha (TNF-α) genetic polymorphisms and endometriosis in 150 Iranian patients suffered this disease. The authors notably found a lower frequency of TNF-α -863C/A allele A among the affected patients in comparison with healthy women, although this difference was not significant by adjusting multiple testing. We deem that the authors should specify, if these patients had peritoneal nodules, ovarian endometrioma/deep infiltrating endometriosis (DIE) nodules or combination of them, since it has been hypothesized that these phenotypes may represent three distinct pathogenetic entities of endometriosis.

Dear editor of International Journal of Fertility and Sterility, We recently read the article by Babaabasi et al. (1) entitled “The Association between TNF-alpha Gene Polymorphisms and Endometriosis in An Iranian Population” recently published in your journal. The aim of this study was to investigate association of some tumor necrosis factor-alpha (TNF-α) gene polymorphisms with risk of suffering endometriosis. The authors notably found a lower frequency of TNF-α -863C/A allele A among the patients with endometriosis compared to the healthy individuals, although this difference was not significant by adjusting multiple testing.

TNF-α is an inflammatory cytokine with a critical role in activating several transcription factors involved in inflammation, such as NF-Kappa-B and c-Jun N-terminal kinases (JNK) (2). Several literatures demonstrated that inflammation and particularly TNF-α may have a contributive role in genesis and establishment of implants of endometriosis (3).

In this letter, we would like to point out a methodological concern of this interesting study: in the “Materials and Methods” section, the authors indicated that analysis of some genotypes in the 5'-untranslated region of TNF-α was done on DNA obtained from peripheral blood samples of 150 Iranian women with confirmed endometriosis. In particular, the polymorphisms were genotyped by using polymerase chain reaction-restriction fragment length polymorphism (PCR-RFLP). We deem that the authors should specify, if these patients had peritoneal nodules, ovarian endometrioma/deep infiltrating endometriosis (DIE) nodules or combination of them, since these three phenotypes of endometriosis may have different pathogenesis. Moreover, it would be of particular interest to know if the risk of having endometriosis in presence of the TNF-α specific -863 A allele was lower, regardless of such endometriosis phenotypes. In fact, we believe that different polymorphisms of TNF-α gene may lead to peculiar transcriptional activity and final protein function, definitively giving a different pathogenic contribution to each endometriotic phenotype (4).

Other genetic polymorphisms have been correlated with specific phenotypes of this disease: for example, it has been reported a significant association of progesterone receptor þ331G/A polymorphism with DIE, suggesting a potential role of this variant in the hormonaldependent invasive behavior of endometrial cells (5). In a population of patients with histologically proved endometrioma without DIE, a specific polymorphism of DNA methyltransferase 3-like (DNMT3L) enzyme, which is responsible for DNA sub-telomeric hypomethylation, has been associated with the presence of endometrioma (6).

Overall, it has been described that nodules of DIE tend to have higher pro-inflammatory microenvironment and oxidative stress, with a more aggressive clinical behavior and different response to conventional therapies in comparison with the other endometriosis phenotypes (7, 8). Hormonal therapies, such as combined hormonal contraceptives and progestogens, should be regarded as the first-line treatment for DIE, considering that they are efficacious, safe and well-tolerated. Gonadotropinreleasing hormone agonists may be employed in women with refractory symptoms of the administration of firstline therapies (8). Surgery has been shown to be highly effective in ameliorating pain symptoms related to DIE implants; however, it may be particularly challenging and the benefits of performing surgical approach in terms of pain improvement should be always balanced with the risk of intraoperative complications (7). Until now, it is not clear if peritoneal endometriosis, ovarian endometrioma and DIE represent three distinct pathogenic entities (4).

Investigations on medical therapeutic approaches for treating endometriosis, represent one of our topics of research: in particular, we recently reviewed the role of experimental drugs targeting inflammation and immune system in this setting (9). Notably, *in vitro* studies have demonstrated that TNF-α is responsible for proliferation ectopic and eutopic endometrial cells as well as the cell adherence within the endometriotic lesions (10, 11). TNFRSF1A and c5N, two human recombinant TNF-α antagonists, have demonstrated to limit growth of endometriotic implants without altering the menstrual cycle in baboons (12, 13). More importantly, as a monoclonal antibody directed against TNF-α and largely used for treating chronic bowel inflammatory as well as rheumatologic disease, infliximab efficiently decreased the size of endometriotic lesions in rats with experimentally induced endometriosis (14). Until now, infliximab has been the only drug evaluated in the clinical setting for endometriosis, blocking TNF-α. However, contrary to the expectations, in a randomized placebo-controlled trial on 21 women, it did not modify number or size of DIE lesions (rectovaginal endometriosis of at least 1 cm in diameter); moreover, pain related to the disease did not ameliorate (15). Although a systematic review by Cochrane underscored that there is not enough evidence to support the use of anti-TNF-α drugs for endometriosis (16), the study of inflammatory-related pathways (including TNF-α polymorphisms) in pathogenesis of endometriosis, and particularly in DIE, appears extremely interesting.

Overall, findings of the study performed by Babaabasi and colleagues (1) are innovative and promising. Nevertheless, we deem that further genetic studies should be performed on this direction, in order to better clarify the role of TNF-α in the multiple aberrant pathways characterizing implants of endometriosis.
